# Role of Mesenchymal Stem Cells on Tonsillar Hypertrophy: An Unexplored Enigma

**DOI:** 10.31729/jnma.7470

**Published:** 2023-01-31

**Authors:** Apar Pokharel, Dharmagat Bhattarai

**Affiliations:** 1 Department of Otorhinolaryngology and Head and Neck Surgery, College of Medical Sciences, Bharatpur, Chitwan, Nepal; 2 Department of Pediatrics, Postgraduate Institute of Medical Education and Research, Chandigarh, India

**Keywords:** *interleukin-4*, *mesenchymal stem cell*, *tonsillar hypertrophy*

## Abstract

Tonsillar or adenoid hypertrophy is a common childhood finding which can cause significant health problems like respiratory infections and sleep apnea. Though normal growth of children is also attributed to such enlargement, infection, environmental pollutants, allergens, and gastroesophageal reflux are proposed triggering factors for tonsillar hypertrophy. While tonsilar enlargement in adults is more associated with malignancy and chronic infections like the human immunodeficiency virus, the immunology of childhood adenotonsillar hypertrophy is less understood. We postulate that upon stimulation, mesenchymal stem cells are found to reduce the secretion of interferon-gamma but increase the secretion of interleukin-4 from activated T cells. Both of these factors inhibit apoptosis in the tonsillar tissue leading to its hypertrophy. Under the umbrella of evidence, it implicates the role of mesenchymal stem cells in tonsillar hypertrophy. However, further longitudinal large studies are needed to validate the proposition.

## INTRODUCTION

Adenotonsillar hypertrophy contributes to significant childhood maladies like recurrent upper respiratory and middle ear infections, jaw and tongue misalignment and obstructive sleep apnea (OSA). Glue ear, difficulty in eating, open bite and reduced appetite are also found to result from tonsillar hypertrophy.^1^

Tonsillar hypertrophy accounts for 80% of cases of obstructive sleep apnea in children.^2-4^ In this article preexisting theories of tonsillar hypertrophy are discussed and a new hypothesis concluding mesenchymal stem cells as the cause of tonsillar enlargement is presented. It can open new frontiers for future research on this common childhood problem.

## PRE-EXISTING THEORIES

In light of the causes of tonsillar hypertrophy, there are few existing theories. However, each of these theories has certain limitations. Some have postulated that previous infections like respiratory syncytial viruses (RSV) and allergies led to changes in neurotrophins, nerve growth factors and inflammatory pathways while others have proposed that innate and inflammatory responses are favoured by tonsillar hypertrophy.^5^ Though there are multiple postulates for common hypertrophic changes in tonsils, the embryo- immunological basis of it is not explored yet. Some of the existing theories on the pathogenesis of tonsillar hypertrophy are analysed here.

1. **Increase in bacterial infection load:** It is implicated to be one of the causes of tonsillar hypertrophy. The size of the tonsil directly depends upon the mean bacterial load. Increased bacterial load causes B and T cells number to increase in the diseased tonsils.^6^ Viral (e.g. RSV) and bacterial infections by Haemophilus influenza and Staphylococcus aureus are described to be associated with lymphoid hyperplasia.^7,8^ Tonsil size is directly proportional to the mean bacterial load per gram of tonsil (colony forming unit (CFU)/ gram). A mean bacterial load of 2.4±2.1x10^4^ CFU/gram was seen in diseased tonsils as compared to 1.6±2.4x10^4^ CFU/gram in normal controls (p<0.01).^6^ A study suggested the presence of Haemophilus influenza and Staphylococcus aureus in the core tissue of tonsillar hypertrophy cases. However, these are also present in recurrent tonsillitis. The exact pathophysiology by which these bacteria behave differently leading to tonsillar hypertrophy is still unexplained.^9^
2. **Allergic reactions:** Allergic response causes the recruitment of basophils in both the early and late phases.^10^ Basophils release interleukin (IL)-4, which inhibits the activity of apoptotic endonuclease. This causes uncontrolled proliferation of tonsillar epithelium.^11^ However, there are studies which show that there is no apparent relationship between allergic disorders and enlarged tonsils.^1,12^
3. **Mutations:** Mutations in the DNA of key cell cycle or apoptosis genes are suggested to cause tonsillar hypertrophy. Changes in DNA methylation can cause modification of gene expression. This can alter many biological processes like cell cycle and apoptosis. Study shows an increased incidence of hypomethylation in patients with tonsillar hypertrophy compared to those with recurrent tonsillitis. DNA hypomethylation is responsible for tonsillar hypertrophy as it can alter the expression of genes related to the cell cycle or apoptosis or activate basophils which leads to an increase in IL-4 level.^13^ However, the exact cause of DNA hypomethylation in tonsillar hypertrophy remains unexplained.

## NOVEL POSTULATE

Our postulate is aimed at alluding that the abnormal stimulation of mesenchymal stem cells (MSC) could be one of the causative factors in tonsillar hypertrophy. There are studies suggesting the presence of mesenchymal stem cells in the tonsils.^14^ Palatine tonsil is a good source of MSC. The tonsillar epithelium is originated from the endoderm and develops through the second pharyngeal pouch. However, the tonsil is invaded by mesodermal lymphoid tissue during fetal development. So embryologically, the tonsil also contains MSC.^15^

1. **Effect of endotoxin on MSC:** TNF-α, an endotoxin (lipopolysaccharide) and hypoxia stimulate human mesenchymal stem cells.^16^ Tonsillar epithelium is under constant exposure to bacterial lipopolysaccharide (LPS) leading to CD14 expression. LPS is binding to lipopolysaccharide (LPS) binding protein (LBP) forming the LPS-LBP complex which then binds to CD14.^17^ CD14 and enhances the ability of mononuclear cells to synthesise tumour necrosis factor-alpha (TNF-α) in response to endotoxin.^18^
2. **Effect of hypoxia on MSC:** Hypoxic condition is the best microenvironment for mesenchymal stem cell proliferation.^19^ Study shows Wharton Jelly-MSC (WJ-MSC) cultured under hypoxic conditions differentiated into a higher number of large and flattened cells. The increased cell size provides an increased surface area to enhance the oxygen diffusion rate.^20^ Thus hypoxia causes both hypertrophy and hyperplasia of the MSC in tonsillar tissues leading to tonsillar hypertrophy. Tonsillar hypertrophy further results in more hypoxia thus leading to a vicious cycle.
3. **MSCs properties:** Tonsillar organs are palatine (oral) lingual tonsils, and pharyngeal (adenoids). All of them are nodular bodies filled with reticular and fibrous mesh filled with immunological cells like macrophages, lymphocytes, mast cells, granulocytes, and MSCs. Germinal centres and follicles are the predominant sites of B cells whereas T cells are found around germinal centres. On the whole tonsils are guards against the microbes entering through oro-nasal epithelial pathways. MSCs are multipotent stem cells initially found in bone marrow but later found in different hematopoietic sites to contribute to hemostasis, regeneration, ageing and wound healing through their ability to differentiate into mesodermal and non-mesodermal tissues. As a result, these MSCs can differentiate into a variety of tissues like bone, cartilage, adipose, muscle tissue and nonmesodermal derived tissues too.^21,22^ These cells are also being studied for engraftment in the site of ischemic injuries to grow new potent organ-specific stem cells.
4. **Interferon-gamma (IFN-γ) and IL-4 in MSCs:** MSC also shares immunosuppressive and immunomodulatory properties. On one hand, MSCs catalyze the complex interaction of antiinflammatory monocytes and regulatory T-cells (Tregs). They also bring functional changes to the wide array of cells namely T cells, B cells, natural killer cells and dendritic cells. They suppress plasmablast and T-cell proliferation. MSCs may contribute to the secretion of programmed death ligands (PDL) resulting in an irreversible reduction of T cell response.^23^ Basically reduced expression of major histocompatibility complex class 2 (MHC-II), CD40, and CD86 on dendritic cells, MSCs contribute largely via the role of IL-6 to reduce CD differentiation and T cell proliferation.^24-26^ Invitro studies performed to assess the immunomodulatory effect of tonsil-derived MSC on T lymphocyte proliferation and specific T-lymphocyte cytokine production showed that the presence of tonsil-derived MSC resulted in decreased proliferation of T cells, decreased interferon-gamma (IFN-γ) production, and increased IL-4 production.^14^ Compared to controls, IFN-γ secretion in T-helper-1(Th1) cells decreased by more than 50% and IL-4 production in Th2 cells increased by more than 300% when they are cocultured with tonsil derived MSCs.^14^
5. **Effects of IFN-γ:** IFN-γ activates cell cycle inhibitors, p21 and p27, which leads to hypo-phosphorylation of the tumour suppressor gene, Rb. This causes suppression of the activity of the E2F family of transcription factors leading to the reduction in the activation of genes (e.g. c-Myc) involved in cell cycle progression. IFN-γ also induces IRF1, a tumour suppressor, and decreases cell survival by decreasing the amount of Bcl2, increasing Bak amounts, reducing mitochondrial function, enhancing the release of Cytochrome c and activation of Caspases. These lead to apoptosis.^27^

IFN-γ synergistically enhances TNF-α induced MSC apoptosis by activating caspase-3. In addition to this, these inflammatory cytokines also induce the autophagy of MSCs. They do this by inducing the expression of Beclin-1. Autophagy downregulates the immunosuppressive function of MSC. Autophagy also inhibits the expression of Bcl-2 via suppression of reactive oxygen species (ROS)/mitogen-activated proteinkinase-1/3 (ERK) pathway. Bcl-2 promotes activation of caspase-3and lead to MSC apoptosis.^12,23^ Therefore, a decrease in IFN-γ promotes the activity of MSC which can lead to tonsillar hypertrophy.

**6. Effects of IL-4:** The inducible lymphocyte Ca^2^+/Mg^2^+ dependent endonuclease (ILCME) is located in the nucleus of the cell. This enzyme is responsible for apoptotic cell death.^29^ IL-4 inhibits the activity of apoptotic endonuclease and leads to tonsillar hypertrophy.^25, 26^

This hypothesis is based upon the findings from various studies conducted on mesenchymal stem cells as discussed above. None of the previous studies has implicated the role of mesenchymal stem cells in tonsillar hypertrophy. On the basis of strong embryo-immunological evidence, we postulate that MSC plays an important role in this pathogenesis. However, this hypothesis needs to be validated through further large longitudinal controlled studies before coming to a concluding point.

## WAY FORWARD

The immunological basis of the pathophysiology of tonsillar hypertrophy is an unexplored arena. Literature on the embryological and immunological links of tonsillar enlargement in children is scarce. The role of MSC in it is completely unexplored. We postulated that repeated tonsillar infection, the release of inflammatory mediators like TNF-α, and a hypoxic environment are responsible for the stimulation of multipotent MSC. Upon stimulation, they cause decreased secretion of IFN-γ and increased secretion of IL-4 from activated T cells. Under these conditions, MSCs become activated which results in tonsillar hypertrophy.
